# Designing an Effective Learning Environment for Language Learning During the Covid-19 Pandemic

**DOI:** 10.3389/fpsyg.2021.752083

**Published:** 2021-10-05

**Authors:** Olkan Betoncu, Funda G. Faslı, Fezile Ozdamli

**Affiliations:** ^1^Department of Computer Education and Instructional Technology, Near East University, Nicosia, Cyprus; ^2^Turkish Preparatory School, Near East University, Nicosia, Cyprus; ^3^Research Center for Applied Linguistics (RCAL), Near East University, Nicosia, Cyprus; ^4^Department of Computer Information Systems, Near East University, Nicosia, Cyprus; ^5^Computer Information Systems Research and Technology Center, Near East University, Nicosia, Cyprus

**Keywords:** m-learning, mobile application, mobile education, distance learning, Turkish as a foreign language

## Abstract

The rapid development in IT in the twenty-first century have along with the COVID-19 pandemic have had effects on the field of education, with the need to use technology for educational purposes arising. Mobile phones, which hold a more important place than computers, also became a part of education. In today's world, it is possible to access educational content without any limitation of time and space. Individuals who desire to learn a new language but cannot do so due to financial difficulties or free time have moved toward mobile learning methods. “YIT101” is a mobile application designed to teach Turkish as a foreign language independent from time and location and targets basic Turkish A1 level course content. This application enables users to access information when needed and to learn Turkish as a foreign language outside the classroom environment independent from a formal instructor, allowing them to save time.

## Introduction

The effects of the crisis that emerged following the World Health Organization announcement on 11 March 2020 regarding the COVID-19 pandemic is still being felt by healthcare organizations and in psychological, financial, educational, and social aspects of life (WHO, 2020). The pandemic affected educational systems worldwide and caused universities and schools to close down (Viner et al., 2020). With the closing of schools, policymakers had to make innovations in order to sustain the education systems (Reimers and Schleicher, 2020).

UNESCO (2020) highlighted the importance of formulating innovative solutions regarding the sustainability of education. Policymakers and educators moved toward producing uninterrupted solutions to continue teaching without any disruptions. This is aimed at making the learning process sustainable (Sintema, 2020). As opposed to the face-to-face educational practices implemented worldwide, the opportunity to use e-learning and m-learning practices as a solution (Babu and Sridevi, 2018) and the main learning source came up (Chun et al., 2021).

Although e-learning was not even on the agenda of many institutions before COVID-19, virtual classrooms and platforms were established globally with the pandemic outbreak. There have been studies on which platforms will be used in education, how the teachers will be supported for e-learning practices (Lockee, 2021), how people without Internet access will be reached (Dhawan, 2020), and how learning will be evaluated (Blume, 2020). Relevant research shows that ubiquitous learning would become the main structure of learning shortly as its functionality will increase with the new technologies and systems (Miah et al., 2020). Mobile learning is a style of education that is independent of time and space (Daud et al., 2021), gives learners access to information at all times, and offers equal opportunities (Kob et al., 2020). Self-learning ability is an important factor in the effective implementation of the learning process through mobile applications. Self-learning abilities can be improved by increasing learners' motivation. Gamification is an education system that aims to increase learner motivation, establish a learner-centered learning setting, involves the student in the process, sets various competitive factors, and achieves learning objectives (Faune, 2021).

Considering foreign language education, it is argued that individuals' interest and concentration rate and their motivation have significant effects on their academic achievements (Denden et al., 2021). If students have fun, get excited, and are motivated during the foreign language learning process (Mustiarini, 2021), the lesson content would become more meaningful and permanent for them.

There have been many scientific studies in recent years on mobile and gamification technologies in foreign language education (Arce and Valdivia, 2020). Based on the given information, it is noted that the use of technology in foreign language education enables learning toward the target language and significant improvements in motivation. It can also be said that the spread of gamified mobile technologies in foreign language teaching would both contribute to the learning process and speed up the process of reaching the desired level.

Additionally, benefitting from mobile technologies in foreign language education is still relevant and current in the field. Mobile-supported learning is mainly observed to contribute to foreigners' listening and speaking education throughout the foreign language learning process (García Botero et al., 2019).

However, there are various challenges in helping foreigners gain the desired basic language skills through the “Turkish as a foreign language” education being offered within the country and abroad (Amalia, 2020). One of the most common examples of such problems is the decrease in students' motivation to learn certain sounds of the language and writing (Sengül, 2014). Another problem is the lack of use of technological innovations in Turkish language teaching.

Various studies from the literature (Zhang and Zou, 2020) highlight the effectiveness of using mobile technologies in foreign language education. The review of relevant literature revealed a limited number of studies on mobile application technologies in teaching Turkish as a foreign language. Also, no studies have been found on gamified mobile applications for teaching Turkish to foreigners in the Turkish Republic of Northern Cyprus. In this context, it becomes evident that there is a need to fill these gaps in the relevant literature.

In light of all this information, this study aimed to develop a mobile e-learning application that would support the gamified ubiquitous learning model by increasing learners' interest and concentration through mobile technologies, helping them learn a language while having fun, and improving their language skills. The study's main purpose was to design and develop a gamified mobile application for university students within the context of the Turkish Language for foreigners.

Learning management systems and e-learning platforms helped to ensure the monitoring of all kinds of tasks regarding learning, (Izumi et al., 2020) had fostering (Ul-Ain et al., 2016) and positive influence on the learning processes (Sáiz-Manzanares et al., 2020).

## Method

As a research model preferred in material development processes, a design-based research method was adopted in this study. The design-based research method concentrates on design and studying all the designed innovations (Design-Based Research Collective, 2003).

The developed designs need to be continuously implemented for students and re-structured based on their needs. The YIT101 application explicitly designed for Teaching Turkish as a Foreign Language is prepared for the Android platform. The Android platform is globally more popular and used more frequently than other platforms due to its flexibility. According to Statita's data from 2021, Android worldwide use was 71,93%. Also, global design principles and design-based research model guidelines were followed while designing and developing applications.

### Data Collection and Analysis

At this stage in the study, first, interviews were conducted with experts who teach Turkish as a foreign language, and the challenges in gaining the four essential skills in language learning were identified. Experts, education technologists, and student opinions were consulted throughout the development process of the application. Semi-structured interview forms were used in order to collect students' views regarding the development of the application. The interviews were conducted with 10 students who participated in the development stage. The researcher conducted the interviews at each stage after the students used the application. The development phase of the application lasted for 12 weeks in total. The following questions were posed to determine students' views:

What are your views regarding the download of the application to your phone and the sign-up process?
What are your views regarding the menu and the content of the application?
What are your views regarding the colors and visuals used in the application?
What are your views regarding the pronunciation activity in the application?
What are your views regarding the listening activity in the application?
What are your views regarding the writing activity in the application?
What are your views regarding the leadership table in the application?

A descriptive research method was adopted to analyze the interview responses used in the study (Strauss and Corbin, 1990). Based on this method, responses were categorized and placed under themes. The application's content was developed according to the information found in the coursebook traditionally used by the students (Istanbul Turkish for Foreigners Coursebook A1).

### Application Development Process

**Figure 1:**
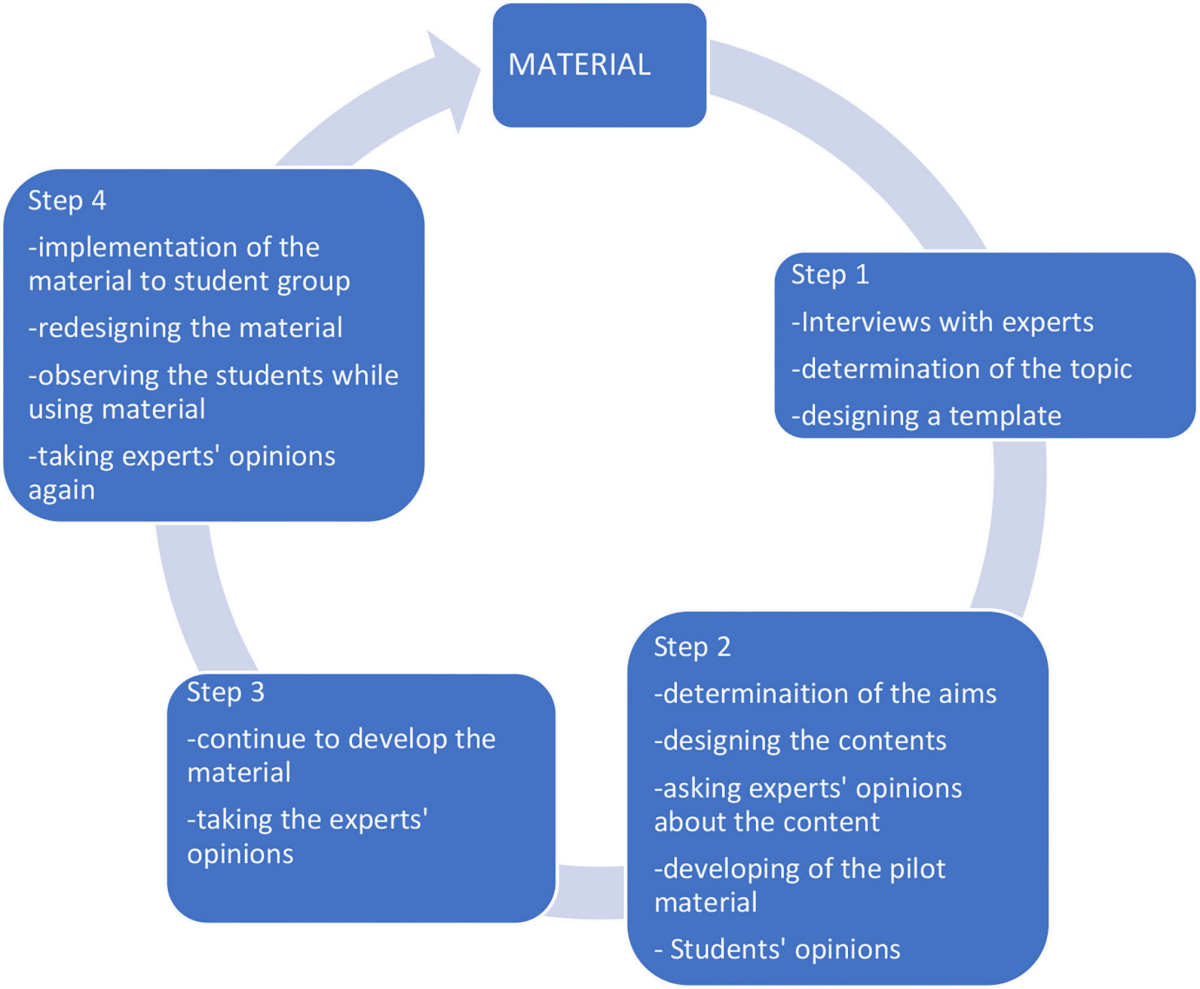
Application development process.

The development process of the application is summarized in Figure 1. It also presents the details of all the steps taken at each stage of the cycle. The application was gradually developed, and regular studies were conducted with the students. Questions emerging at the end of the process were analyzed, the then the application was improved and presented to students to use again.

#### Mobile Application Development Tools

YIT101 application was developed on a device with Windows 10 operating system. Firebase database technology offered by Google was used on the side of the server to store the data. The exact device test of the application with the devices in all dimensions of the emulator was done using a mobile device with an Android operating system.

## Results

A needs analysis was conducted before the design and implementation processes of the study in order to determine which topics and purposes the Teaching Turkish as a Foreign Language application would address. An interview form consisting of 10 questions was implemented to the experts who teach Turkish to foreigners in Northern Cyprus and educational technologists.

The needs analysis results indicated that experts generally believe that the YIT101 application would effectively support international students while learning Turkish and that the experts are inclined to take an active part in this process. When experts' use of mobile devices for educational purposes was examined, it was observed that they were mainly used for material sharing (instructional videos or documents), dictionary use, and communication.

The field experts reported that, due to the limitations of the current Turkish language education practices, educational materials developed for this purpose would be beneficial. The application should be designed for the basic needs and characteristics of the learners. They also stated that there are applications for the Turkish language on Google Play but these are not suitable for those learning it as a foreign language. Educational technologists highlighted that the Turkish as a Foreign Language Education (YIT101) application needs to have features that would increase learners' motivation. The field experts who are teaching foreigners the Turkish language through distance education listed the topics they need help with the most at Turkish A1 level and thus should be targeted in the application as The Alphabet, Sound Formation, Meeting and Greeting, Plural Form, Question Tags, Personal Pronouns, Demonstrative Pronouns, Possessives, and Numbers.

### Step 1

As stated before, the mobile application was developed based on the regular feedback received.

During this process, the content and usability of the application are evaluated based on field experts' opinions. Interviews were conducted with the field experts, and their views were collected regarding the mobile application. As a result of the interviews, it was decided to include gamification elements into the Turkish education application to increase learners' motivation. Screens and contents for all topics were designed, and the object used on the designed pages were decided with the field experts.

Based on the expert opinions, the most suitable mobile application was written over the AndroidStudio platform using Kotlin and Java software languages. YIT101 application is an Internet-based application; thus, it requests permission to access the Internet during the setup and later when using the application. It informs the user with a warning notification when the device has no Internet connection, and the application cannot be used when the device has no Internet access. Then, the users are required to give microphone access permission to use the pronunciation and speaking activities of the application. This permission is needed for Google's Text-to-Speech API. SplahsScreen from the screen was used while loading the needed data during the opening of the application.

#### Revisions After Feedbacks

- From the gamification elements decided during the interviews with the field experts and educational technologists, features of the leader board and points were added to the application.

**Figure 2:**
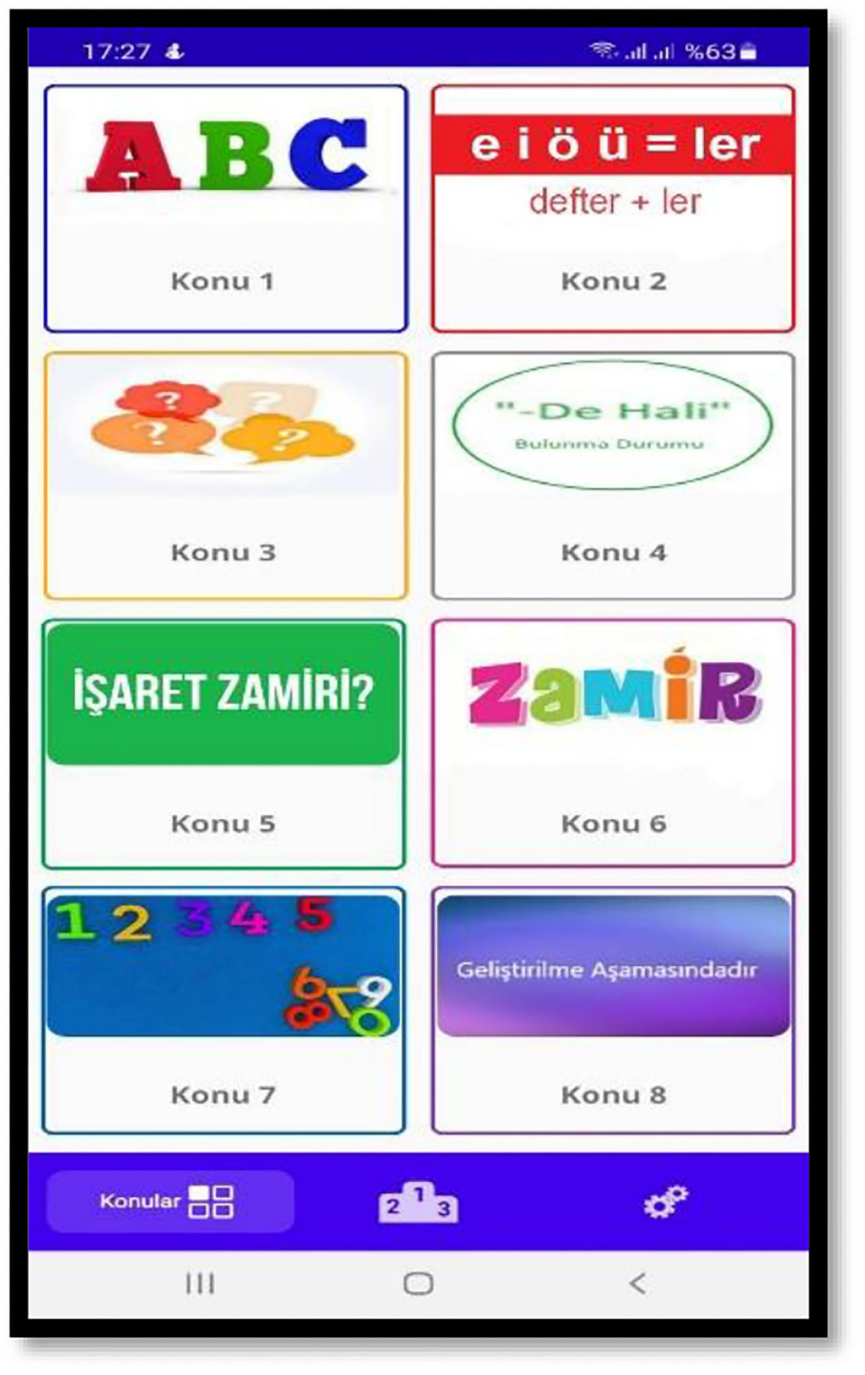
Main menu of the application.

As shown in Figure 2, visuals to help understand the content were added to the menu, which included the content.

Figure 2 shows the main menu of course content prepared by taking the topic and curriculum into account. The users can begin with a topic of their choice and learn at their own pace. Also, they can check which level they are at with the level feature added as another gamification element. The two icons located at the bottom are for the leader board and settings menu, respectively. The application logo was resized and set for all Android versions. Google Crashlystic was used to check and manage the potential errors during the use of the application.

### Step 2

The success of a mobile application depends on to what extent the users internalize all features of the application and how much benefit they receive from these features. A well-designed user interface would both help internalize the topic earlier, and increase the application-user interaction (Invonto). There is visual and audio guidance in addition to the written guide to help students learn. Nevertheless, the students reported that clearing the scoreboards at specific intervals and making the main menu more interesting would be good improvements.

#### Revisions After Feedback

**Figure 3:**
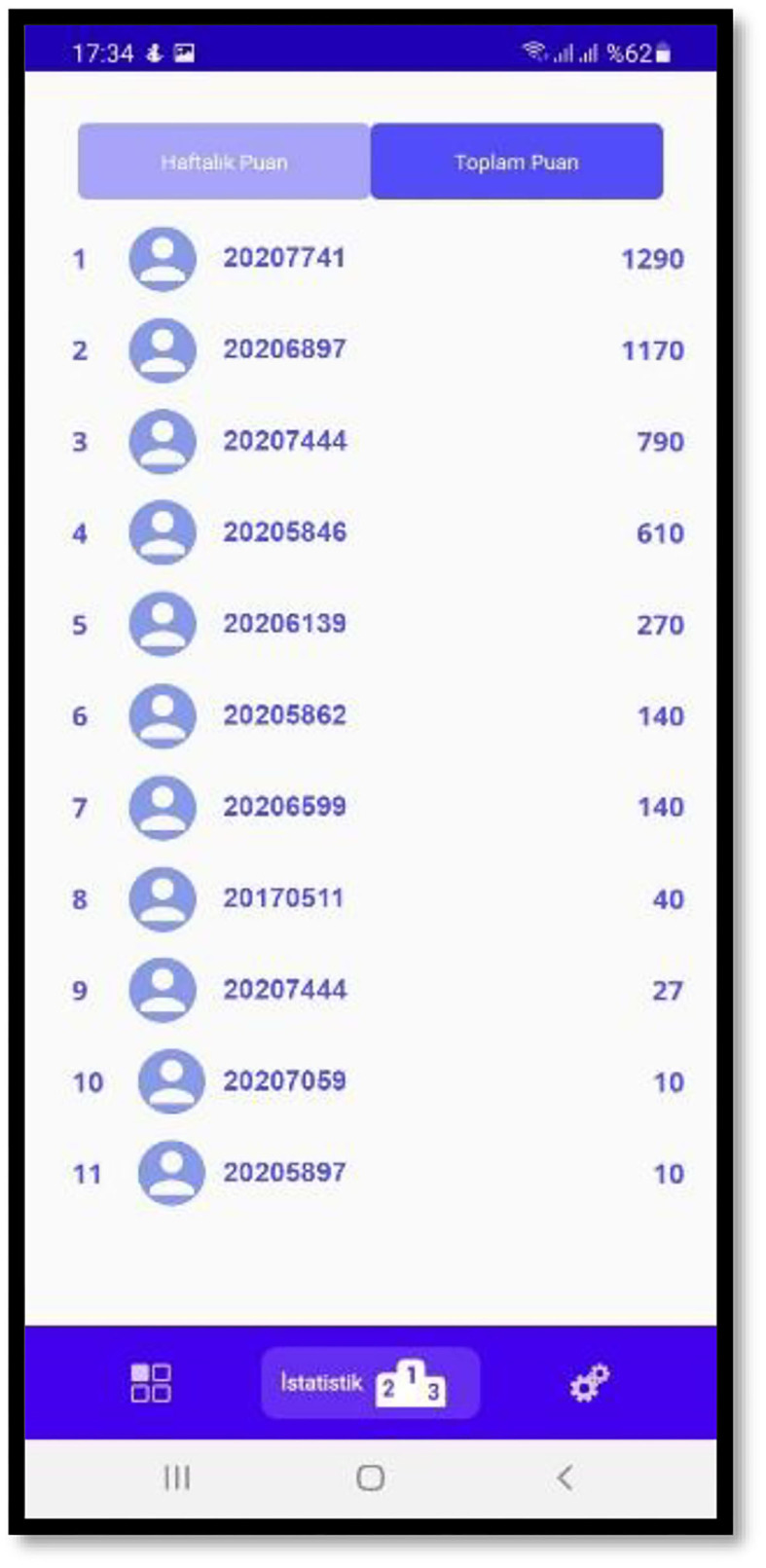
Score board.

**Figure 4:**
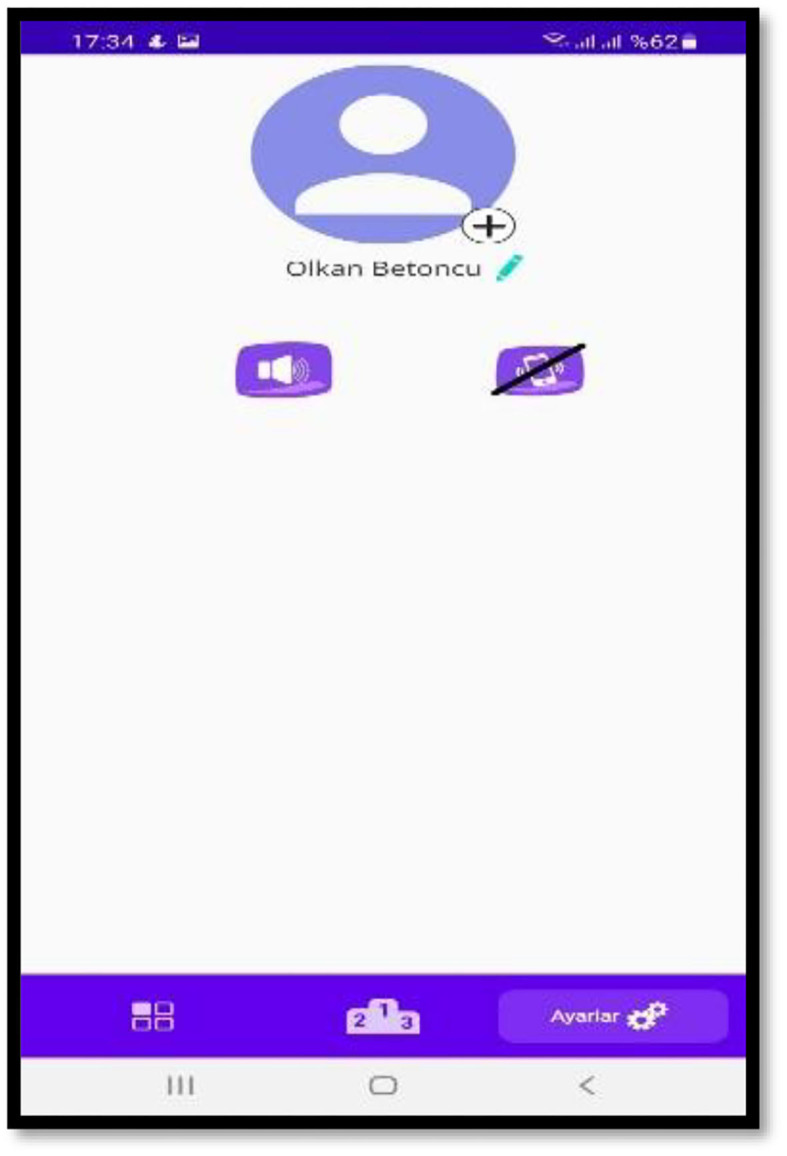
Settings menu.

- Scoreboards are cleared weekly via the Google Cloud function (Figure 3). The application includes users' weekly scores table. This part of the scoreboard is cleared once a week to increase the competitiveness feature of gamification. There is also a list of the total scores received by the users. The full scoreboard is not cleared in any way. It presents the accumulated points received by all the correct answers given by the users.
- Pronunciation check activity (Figure 4) was added for all topics. This helps learners, in the pronunciation section of the application, to check learners' pronunciation of the words and sentences taught at the end of each activity.
- Text-to-speech feature was added to help learners listen to the audio of the texts.
- Putting the dialogue in the correct order activity was added with the drag and drop technique. This added variety to the application and increased the number of the acknowledged scientific methods used in language learning.

### Step 3

Once the students touch the correct answer, a visual reinforcer such as “correct” or “bravo” appears on the screen along with a verbal reinforcer. However, as the field experts reported that the verbal reinforcer would distract the learners, they suggested adding a sound effect. To ensure learning, the wrongly answered questions are presented to the students at the end of the activity again so they learn the correct answer.

#### Revisions After Feedback

- Verbal feedback was replaced with sound effects.
- All topics were fostered with visuals.

### Step 4

A significant part of the application was completed and downloaded to the learners' phones at this stage. The students were asked to use the application for two weeks as part of the YIT101 course. The final opinions were collected from the students and the field experts at the end of the two weeks. The feedback from the students showed that the students with e-mail accounts other than Google experienced problems while entering the system as all users are required to sign in with their Google accounts. The students also suggested that the gamification elements create a competitive setting and continue learning to beat their friends.

#### Revisions After Feedback

- The students were given the option of using a different e-mail address to enter and sign in to the application. Hence, individuals without Google accounts could also easily use the application.
- Settings window (Figure 4) was added to the application. The users were allowed to turn off the sound effects used for correct and wrong answers and the on-touch vibration of the application from this window. Thus, the users could study in settings when they need to be quiet and save on battery life during more extended study periods.
- It was decided to use customized sounds in the listening and other sections; so, the sounds were changed.

Upon completing the necessary evaluations and arrangements, the final version of the application was uploaded to Google Play.

## Discussion and Conclusion

Foreign language learning is ensured by improving reading, writing, listening, and speaking skills (Darancik, 2018). A variety of mobile applications, either for reasonable prices or free, are designed to improve these skills. This study aimed to develop a gamified mobile application to foster individuals' learning of Turkish as a foreign language. The education of students learning Turkish as a foreign language necessitates the use of applications that are tailored for individual learner differences, current student profile, supported by technology, create independence of time and space, help students access information at all times, and give equal opportunities in education (Kob et al., 2020). Design-based research methods were preferred at the first stage of the study, the software development phase. Based on user opinions, it was concluded that the design of the YIT101 application, which was developed through a design-based research process, was found to be satisfying, user-friendly, and filled with valuable content. Special attention was paid to making the application easy to understand and use in its visuals (colors, drawings, text, photographs, etc.). Although the dimensions of the objects are different from real life, they should be used in an appropriate and understandable scale on the screen (Çiloglu et al., 2021); otherwise, it may result in a conflict on the part of the students. In this regard, it is noted that easy, understandable, and simple materials need to be developed (Evren, 2016). As the building block of foreign language education, vocabulary learning has become rather practical and easy with mobile language learning applications (Kacetl and Klímová, 2019). An important method in ensuring a fun and continuous learning is to gamify the learning process. Thus, gamified mobile applications have an important place in language education (Sen, 2019). The application developed based on typing and writing would then increase the memorability of the learned vocabulary by using reading, writing, listening, and speaking features together. The suggested application educated the user with more than more sensory organs and taught longer-term vocabulary (Kuşçu, 2017), which increased the variety offered and improved memorability.

When the process outcomes were reviewed after developing the material, different activities such as drag and drop, multiple choice, pronunciation check, and ordering the dialogue were added to the activities as a variety to the written or typed activities. This enabled users to stimulate reading, writing, speaking, and listening, as the four basic language skills. Another decision made within the application was to involve gamification elements in order to increase students' motivation. Scoring and leadership list systems were used in the application to increase and achieve the competitive factor as one of the gamification elements. It is believed that this would increase the competition among students and encourage long-term, more permanent learning. When continuing to review the outcomes, it is seen that more accurate and understandable reinforcers need to be given to the users. If the reinforcers given at the end of the activities are not suitable, they might distract the learners and not trigger the necessary motivation. It was thought that presenting learners with the questions they could not answer correctly only once would not sufficiently reinforce the topic and so aimed to be reinforced by asking them again at the end of the activity. It was concluded that visuals and different sound effects as reinforcers would be crucial for motivation. One of the most basic principles of mobile application, without a doubt, is to give learners freedom (Altuntaş, 2017). In this regard, there is a need for settings panels to help users turn off the sound and vibration, considering that they might want to or in settings where they cannot use the application with sound. This way, the learners can study in a setting where they need to be quiet and save on battery life to study for more extended periods. If the designed applications would require the users to set up profiles, they need to consider using different e-mail address accounts. The users should be able to set up their accounts with different e-mail addresses.

In conclusion, this study emphasizes the issues to be considered in terms of content, usability, and interaction while developing interactive mobile application materials for students learning Turkish as a foreign language. The international literature includes many studies on mobile technologies in foreign language education, especially English language education. However, the number of practices and studies for the Turkish language within TRNC and Turkey was insufficient. It is hoped that the developed application would contribute to this gap and provide guidance for future studies.

## Data Availability Statement

The raw data supporting the conclusions of this article will be made available by the authors, without undue reservation.

## Ethics Statement

The authors assert that all procedures contributing to this work comply with the ethical standards of the relevant national and institutional committees on human experimentation. All participants gave written informed consent in accordance with the Declaration of Helsinki. The study was approved by the Scientific Board of Near East University. The patients/participants provided their written informed consent to participate in this study.

## Author Contributions

OB designed and carried out the study and contributed to the analysis of the results and to the writing of the manuscript. FF designed and carried out the study, collected data, and contributed to the writing of the manuscript. FO contributed to the analysis of the results and to the writing of the manuscript. All authors contributed to the article and approved the submitted version.

## Conflict of Interest

The authors declare that the research was conducted in the absence of any commercial or financial relationships that could be construed as a potential conflict of interest.

## Publisher's Note

All claims expressed in this article are solely those of the authors and do not necessarily represent those of their affiliated organizations, or those of the publisher, the editors and the reviewers. Any product that may be evaluated in this article, or claim that may be made by its manufacturer, is not guaranteed or endorsed by the publisher.
